# The Potential of Black Soldier Fly (*Hermetia illucens* L.) Larvae in Whole Wheat Bread Production: Effects on Physicochemical, Antioxidative, and Sensory Properties

**DOI:** 10.3390/foods14213686

**Published:** 2025-10-29

**Authors:** Anna Draszanowska, Marta Czarnowska-Kujawska, Beata Paszczyk, Małgorzata Starowicz, Magdalena Anna Olszewska

**Affiliations:** 1 Department of Human Nutrition, The Faculty of Food Science, University of Warmia and Mazury in Olsztyn, Słoneczna 45f, 10-718 Olsztyn, Poland; 2 Department of Commodity Science and Food Analysis, The Faculty of Food Science, University of Warmia and Mazury in Olsztyn, Plac Cieszyński 1, 10-726 Olsztyn, Poland; 3 Chemistry and Biodynamics of Food Team, Institute of Animal Reproduction and Food Research of Polish Academy of Sciences, Trylińskiego 18, 10-683 Olsztyn, Poland; m.starowicz@pan.olsztyn.pl; 4 Department of Food Microbiology, Meat Technology and Chemistry, The Faculty of Food Science, University of Warmia and Mazury in Olsztyn, Plac Cieszyński 1, 10-726 Olsztyn, Poland

**Keywords:** whole wheat bread, black soldier fly larvae, physicochemical properties, antioxidative properties, color, texture, sensory analysis

## Abstract

This study explores the incorporation of black soldier fly larvae (*Hermetia illucens* L.; HI) into whole wheat bread to enhance the nutritional quality of this staple food. Two inclusion levels were tested: 10% (HI10) and 30% (HI30). The larvae meal exhibited high protein (37%) and lipid levels (48%) along with a high microbiological safety level. Enriched breads demonstrated enhanced nutritional quality, with a 21–57% increase in protein content, a 3.7–6.0 times increase in lipid content, and significant increases in mineral content, particularly 1.7–3.2 times more calcium among macroelements and 1.3–2 times more manganese among microelements. However, breads contained lower levels of monounsaturated (MUFAs) and polyunsaturated fatty acids (PUFAs) compared to saturated fatty acids (SFAs), with high levels of lauric acid reaching 38–47%. Although significant, the breads exhibited modest increases in total phenolic content (TPC) and antioxidant activity (PCL), with levels 1.1–1.3 times and 1.1–1.2 times higher, respectively. As the proportion of larvae increased to 30%, the lightness, yellowness, and chroma of the bread crumb significantly decreased. The crumb became harder, reducing its springiness, but chewiness remained similar in HI0 and HI10. Sensory evaluations showed no significant differences between HI0 and HI10 for 90% of attributes. The study concluded that incorporating HI larvae into breads enhanced their nutritional qualities while maintaining physical and sensory parameters satisfactory at a 10% addition.

## 1. Introduction

The demand for sustainable and nutritious food sources has emerged as a critical challenge in our efforts to feed millions of people worldwide. This need is particularly pressing given that the global population is projected to rise to 8.5 billion by 2030 and 9.7 billion by 2050 [1,2]. Traditional food production methods consume many resources and greatly impact the environment, leading to soil degradation, deforestation, biodiversity loss, excessive water usage, and increased greenhouse gas emissions [3]. In recent years, the food industry has begun to view insects as a viable future food source [4] because edible insects are one of the alternative protein sources that can improve human nutrition [5]. Importantly, insects reproduce rapidly, require minimal land space, and are highly nutritious, particularly in terms of protein [6,7,8]. In developed countries, several barriers prevent the acceptance of insects as a food source. These include food neophobia, which is a fear of trying new foods, and feelings of disgust due to a general unfamiliarity with consuming insects [9,10]. Research shows a negative relationship between food neophobia and the willingness to consume insects [11]. In an Australian study, 87.6% of individuals with high food neophobia had never eaten insects, compared to 42.1% of food neophiliacs [12]. Similarly, a study conducted in the United States found that 67% of respondents who had never eaten insects found the idea disgusting, while only 35% of those who had tried them felt the same [13]. Furthermore, a survey that included participants from Belgium, China, Italy, Mexico, and the USA found that both food neophobia and disgust decrease the likelihood of consuming insects, whether whole or processed [14]. Additionally, cultural perceptions [9,15] and a strong attachment to traditional meat sources also contribute to the resistance towards insects [16].

Exposure to insect-based foods is a key factor influencing the perception of entomophagy and the familiarity of these products among consumers [12,13]. Using milled insects in bakery products presents an attractive option, as it effectively removes visual associations typically connected with insects [17]. Research by Orkusz et al. [18] and Herbert and Beacom [9] shows that consumers are more receptive to incorporating edible insects into familiar baked goods. Factors such as cost, ease of preparation, convenience, and shelf life also contribute to the increasing popularity of these products [19]. Flours derived from edible insects have gained attention for their potential use in various baked goods, including bread [20], muffins [21], cakes [22], pasta [23], and snacks [24]. Among the various insects, the yellow mealworm (*Tenebrio molitor*) is the most commonly utilized due to its high protein content, dietary fiber, unsaturated fats, and essential minerals.

While traditionally used as animal feed, black soldier fly (*Hermetia illucens* L.) larvae are now receiving considerable attention as a promising novel food ingredient for human consumption. Black soldier fly is a saprophytic insect that has gained attention from both the scientific community and industry due to its ability to feed on various types of organic waste, its rapid growth, and its minimal environmental impact [25,26]. The larvae have several advantages over other edible insect species [27]. They contain a high crude protein content that exceeds 40% [28] and are rich in essential micronutrients, including Ca, Cu, P, K, Mg, Na, Zn, Fe, Mn, and Zn. Additionally, they provide significant amounts of macronutrients, such as proteins, fats, and fibers, as well as essential fatty acids [29,30,31]. Furthermore, the amino acid profile of the larvae meets the World Health Organization (WHO) standards, making them a potentially excellent alternative protein source for human consumption [32].

While bread is a staple in many diets and primarily serves as a source of carbohydrates, it is typically low in nutrient density. Hence, enriching bread with insects could significantly enhance protein, fat, and dietary fiber content [33]. This approach may also effectively improve nutrient intake for specific populations without requiring significant changes to their diets [34]. However, incorporating insects can impact gluten formation and overall product quality [35]. A few recent studies have investigated the effects of black soldier fly larvae on the quality of tortilla chips [36], white bread [37], and cookies [38] but have not thoroughly looked into the health-promoting nutrients, such as minerals, fatty acids, or antioxidants. Additionally, they did not test all possible bread formulations or insect additions. One possible approach is to use larvae as a substitute for whole wheat flour. Both share similar darker color characteristics, which could help eliminate any visual association with the black soldier fly in the product. It also allows for experimentation with different inclusion levels. Given that whole wheat bread has a denser structure than white bread, it may help mask the effects of insects on textural properties, especially since including *H. illucens* results in a lower volume of wheat bread compared to other insects [37], which reinforces the suitability of using whole wheat flour for this purpose. This study investigates the properties of bread made with dried black soldier fly larvae by replacing whole wheat flour with insect meal at 10% and 30% to observe the effects of higher addition levels. We are examining the meal itself and its impact on protein and fat content, mineral levels, fatty acid profiles, and antioxidative properties, as well as textural, color, and sensory characteristics of the bread.

## 2. Materials and Methods

### 2.1. Materials and Sample Preparation

**Figure 1:**
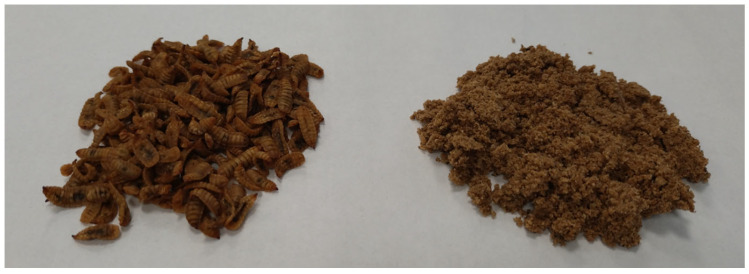
Dried black soldier fly larvae before milling (left) and after milling (right).

The ingredients for the bread were purchased from a local discount store in Olsztyn, Poland. Dried black soldier fly larvae (*Hermetia illucens*; HI) were sourced from a small-scale local insect breeder. They were reared in a small batch within a food-grade plastic container with a warm, moist environment of 25 °C and 60% humidity, and primarily fed wheat bran, with the addition of fruits and soft vegetables like tomatoes, cucumbers, zucchini, and pumpkins. They were harvested within 14 days, then frozen (−20 °C for 48 h) and dried hot-air in an oven for 12 h at 65 °C (DS-80-3O; Dasol Scientific Co., Ltd., Hwaseong, Republic of Korea). The dried larvae were stored in zip-lock bags in a dark environment. The dried black soldier fly larvae were ground using a multifunctional Robot Coupe^®^ device (R 301 ultra D, Vincennes, France) (Figure 1). Before use, the insects were tested for nutritional value and microbiological safety.

**Figure 2:**
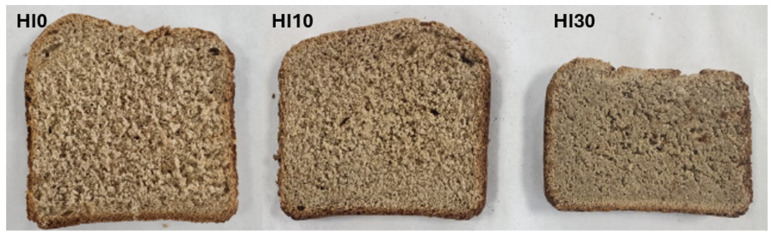
Breads made with different amounts of dried black soldier fly larvae: HI0 (0%; no insect meal), HI10 (10%), and HI30 (30%).

All ingredients were combined using a Kenwood planetary mixer (KWL90.244SI, Havant, UK). In the initial stage, a bread recipe was developed by substituting a portion of whole wheat flour with the meal made from black soldier fly larvae. A control sample of bread without HI (0%), as well as breads fortified with 10% and 30% HI, were prepared using an automated bread machine (Tefal PF6118, 1600W, Is-sur-Tille, France). The tested bread formulas are presented in Table 1. After the baking process was completed, the loaves were allowed to cool at room temperature in the dark. Some of the loaves were then ground in the Robot Coupe^®^ for further analysis. The three types of baked breads are shown in Figure 2.

### 2.2. Proximate Composition

The breads were analyzed following the procedures established by the Association of Official Analytical Chemists. The moisture content was as outlined in AOAC procedure No. 934.01 [39]. Crude protein content was assessed using the Kjeldahl method (AOAC No. 2001.11) [40]. Crude fat content was measured using the Soxhlet method (AOAC No. 991.36) [41] with pure petroleum ether (No. 945.16) [42].

### 2.3. Minerals

#### 2.3.1. Reagents and Materials

Hydrated lanthanum chloride (LaCl_3_•7H_2_O; CAS no. 10025-84-0), used in minerals analysis, was obtained from Merck (Darmstadt, Germany), while ammonium molybdate VI (CAS no. 12054-85-2), sodium sulfate IV (CAS no. 7757-82-6), and hydroquinone (CAS no. 123-31-9) were purchased from “POCH” S. A. (Gliwice, Poland). Other chemicals used in the experiments were of at least analytical grade and were purchased from Merck (Darmstadt, Germany) and “POCH” S.A. (Gliwice, Poland).

#### 2.3.2. Determination of Minerals

Minerals were analyzed as described in detail in previous study of Draszanowska et al. [43]. In brief, the acetylene–air flame emission technique was employed for the determination of potassium (K) and sodium (Na) using an atomic absorption spectrometer (Thermo iCE 3000 Series, Waltham, MA, USA). Calcium (Ca), Magnesium (Mg), zinc (Zn), iron (Fe), manganese (Mn), and copper (Cu) were analyzed using a flame atomic absorption spectrometer (Thermo iCE 3000 Series; Madison, WI, USA). Measurements were conducted at the following wavelengths: K (766.5 nm), Na (589.0 nm), Ca (422.7 nm), Mg (285.2 nm), Zn (213.9 nm), Fe (248.3 nm), Mn (279.5 nm), Cu (324.8 nm). Device parameters such as acetylene, air, electronics and optics were adjusted for each mineral to provide maximum absorption. The determination of phosphorus (P) was performed using a colorimetric method. After converting ammonium molybdate (VI) into phosphomolybdates, the reduction to phosphomolybdenum blue was carried out with the use of hydroquinone and sodium (IV) sulfate. Absorbance was measured using a KRÜSS-OPTRONIC spectrophotometer (Hamburg, Germany) at 610 nm.

### 2.4. Determination of Total Phenolic Content (TPC) and Antioxidant Activity

#### 2.4.1. Extract Preparation

A total of 1 mL of an 80% methanol solution was added to 100 mg of the sample. The solution was then extracted using an ultrasound-assisted method as detailed by Starowicz et al. [44]. Each mixture was vortexed for 30 s, sonicated for 30 s, and subsequently centrifuged for 5 min at 5000× *g* and 4 °C. This extraction process was repeated five times. After each iteration, the residue was resuspended with an additional 1 mL of fresh 80% methanol. The supernatants were combined and collected in a 5-mL volumetric flask. Three independent extractions were performed for each sample. The extracts were stored at −80 °C prior to further analysis of the total phenolic content (TPC) and antioxidant activity.

#### 2.4.2. Total Phenolic Content (TPC)

The total phenolic content (TPC) was measured using a microplate reader (Infinite M1000, Tecan, Männedorf, Switzerland) according to the method by Starowicz et al. [44]. In brief, 0.25 mL of a 20 mg/mL extract was mixed with 0.25 mL of Folin’s phenol reagent, 0.5 mL of saturated sodium carbonate (Na_2_CO_3_), and 4 mL of distilled water. After incubating the mixture at room temperature (21 °C) for 25 min and centrifuging it at 2000× *g* for 10 min, the absorbance of the supernatant was measured at 725 nm. TPC results are reported as milligrams of gallic acid equivalents per 100 g of dry matter (d.m.).

#### 2.4.3. Antioxidant Activity

Antioxidant activity was assessed using the photochemiluminescence method (PCL) with the PHOTOCHEM^®^ apparatus (Analytic Jena, Jena, Germany). The protocols for measuring the Antioxidant Capacity of Water-soluble substances (ACW) and Liposoluble substances (ACL) were employed to evaluate the extracts’ scavenging activity against superoxide anion radicals (O_2_•−). The luminal reagent, Trolox stock, and working solutions were prepared according to the manufacturer’s instructions. Extracts were added at concentrations that ensured the resulting luminescence fell within the standard curve limits. In both modes, Trolox served as the standard, with concentrations ranging from 0.25 to 1.00 nmol. To validate the measurements, two blanks and a set of diluted Trolox solutions were run, as recommended in the PCL protocol. The results obtained were expressed as µmol of Trolox equivalents (TE) per 100 g of dry matter (d.m.).

### 2.5. Determination of Fatty Acid Profile (FAs)

#### 2.5.1. Fat Extraction

The composition of fatty acids in the fat extracted from the samples was determined using the method described by Draszanowska et al. [43]. The fat from the meal and bread samples was extracted using the Folch method [45].

#### 2.5.2. Fatty Acid Profile

Fatty acid methyl esters were prepared from total lipids using the Peisker method, which involves a solution of chloroform, methanol, and sulfuric acid in a volume ratio of 100:100:1 [46]. The fatty acids in the methyl esters of each sample were analyzed by gas chromatography (GC) using a 6890N gas chromatograph (Agilent Technologies, Santa Clara, CA, USA) equipped with a flame ionization detector (FID). The analysis was conducted on a 30-m Stabilwax^®^ (Supelco, Bellefonte, PA, USA) 10 capillary liquid column with a thickness of 0.25 μm and an internal diameter of 0.32 mm. The separation parameters were set as follows: detector temperature at 250 °C, injector temperature at 230 °C, and column temperature at 195 °C. Helium was used as the carrier gas, flowing at a rate of 1.5 mL/min. A standard mixture of 37 components, Supelco FAME Mix (Supelco, Bellefonte, PA, USA), was utilized for the identification of fatty acids. All analyses were performed in triplicate.

### 2.6. Texture Profile

Texture Profile Analysis (TPA) was conducted on breads using a TA.TXplus (Stable Micro Systems Ltd., Godalming, UK). To perform the measurements, a sample of the soft part of the bread was placed on a fixed bottom plate. Each sample underwent a two-cycle compression lasting 5 s, with a 50 kg load cell. The compression reduced the sample to 50% of its initial height using a P/100 platen with a 100 mm diameter, which moved at a constant speed of 1 mm/s, with a trigger force of 5 g. The specimens analyzed were cubes of crumb measuring 30 mm × 30 mm × 30 mm, with ten specimens evaluated for each treatment. The texture profile analysis aimed to determine texture properties, including hardness, springiness, cohesiveness, gumminess, and chewiness.

### 2.7. Color Measurement

The color of bread samples, including both the crust and crumb, was measured using a CR-400 colorimeter (Konica Minolta Sensing Inc., Osaka, Japan). This measurement focused on three parameters: L*, a*, and b*, with a standard observer at 2° and the illuminant set to D65. L* represents luminance or lightness, ranging from 0 (black) to 100 (white). The parameter a* varies from green (−) to red (+). The parameter b* ranges from blue (−) to yellow (+). The colorimeter was calibrated using a white ceramic plate provided by the manufacturer. Color space L*a*b* coordinates, which include lightness, redness, and yellowness, respectively, were recorded from ten randomly selected locations on the surface of both the crust and crumb of the bread. Based on these measurements, chroma (C*) and hue angle (h°) were calculated using specific formulas:

(1) $$C*\;=\;\sqrt{a{*}^{2}+b{*}^{2}}$$

h° = arctg(b*/a*) × (360°/2 × 3.14)(2)

Brownness index (BI) was calculated as follows [47]:

BI = 100 (*x* − 0.31)/0.172 (3)

where

*x* = (a* + 1.75 L*)/(5.645 L* + a* − 3.012 b*)

### 2.8. Sensory Analysis

The sensory evaluation took place in a specialized laboratory with individual assessment booths. A panel of 10 trained assessors, aged 20 to 40, from the Faculty of Food Science at the University of Warmia and Mazury in Olsztyn, Poland, participated. All panelists demonstrated sensory sensitivity as per ISO 8586:2012 [48] and had prior experience with insect-based products. They provided informed consent for the study, approved by the Ethical Committee (KEBN/8/2025), and were free from food neophobia. Three 10 mm thick slices of each bread type were randomly placed on labeled porcelain plates. Panelists scored eleven sensory attributes on a scale from 0 to 10. The sensory attributes evaluated included the following: overall appearance, aroma and taste acceptability, overall acceptability (where 0 indicates “not acceptable” and 10 indicates “very acceptable”), intensity of insect and foreign aromas and tastes (0 for low intensity and 10 for very high intensity), as well as crumb porosity and elasticity (0 for low quality and 10 for very high quality).

### 2.9. Statistical Analysis

Data analysis was performed using TIBCO^®^ Statistica™ ver. 13.3 (TIBCO Software Inc., Tulsa, OK, USA), where one-way ANOVA was applied to test the differences between breads at *p* < 0.05.

## 3. Results and Discussion

### 3.1. Insect Meal Characterization and Chemical Composition of Bread

#### 3.1.1. Nutritional Value and Microbiological Safety of Insect Meal

Under rearing conditions and post-harvest processing provided, the black soldier fly larvae contained 1.98% water, 36.70% protein, and 47.70% fat with 3.75% ash and dietary fiber at 5.71%, similar to previous studies by, e.g., Saraswati et al. [36] (Table 2; Table S1). As the popularity of insects continues to rise, concerns about their safety are also increasing. This has prompted an investigation into the microbiological safety of *H. illucens*. A study by Siqueira Galvão Novo et al. [37] found elevated levels of yeast and mold in HI, underscoring the need for improved manufacturing practices and the establishment of microbiological quality control in insect rearing. However, the microbial counts for black soldier fly larvae met the European Union’s safety criteria for edible insects in all tests (Table S2). This suggests that the manufacturing practices used for rearing the larvae used in this study adhere to the highest safety standards. While the microbiological safety of the HI meal is confirmed, other safety aspects, such as the potential presence of allergens, still require further investigation. Nevertheless, these findings are promising and help alleviate some safety concerns.

#### 3.1.2. Proximate Composition of Bread

The primary ingredients in most gluten-containing baked goods are wheat flour and water, which are energy-dense but not very nutritious. The nutritional value of these bakery products can be enhanced by adding powder made from edible insects [33]. The water, protein, and fat content of each bread formulation is presented in Table 2. The water content in the bread increased significantly with the proportion of black soldier fly larvae (*p* < 0.05; 30%). This increased moisture content may be attributed to the high-fat content of the meal, which inhibited water evaporation during baking [49]. Additionally, the increase in moisture content associated with the HI addition can be linked to its elevated protein content. According to Nandiyanto et al. [50], protein has a strong ability to bind water, indicating that a higher protein content leads to a greater water absorption capacity. In contrast, Saraswati et al. [36] found that tortilla chips with 5% larvae flour had the highest moisture content at 2.25%, while those with 20% had the lowest at 1.84%. This shows that more larvae flour leads to lower moisture levels, as frying releases free water from the material, thus the moisture is highly dependent upon the processing methods.

Adding dried black soldier fly to bread increased both protein and fat content (Table 2). Breads containing 30% black soldier fly achieved a protein content of 15.57% and a fat content of 1.81%. This represents an increase of 56.64% for protein and 503.33% for fat, which are 1.5 times and 6 times the original amounts, respectively. Additionally, breads with 10% black soldier fly experienced a protein increase of 21% and a fat increase of 267%. Similarly, Wrasiati et al. [38] demonstrated that increasing amounts of HI in cookies resulted in higher moisture, protein, and fat levels. The highest increases were observed with the addition of 25% insect meal, yielding increases of 51%, 194%, and 107%, respectively. For comparison, Siqueira Galvão Novo et al. [37] reported that supplementing bread with 15% black soldier fly led to increases of 48% and 27% in protein content, and a 21-fold and 9.5-fold increase in fat content when compared to wheat control and whole wheat control, respectively. Saraswati et al. [36] also indicated that greater additions of larvae flour (ranging from 5% to 20%) resulted in higher protein and fat content in the resulting tortilla chips.

### 3.2. Minerals Analysis

#### 3.2.1. Mineral Composition of Insect Meal

The mineral composition of breads, including the dried black soldier fly larvae meal used for supplementation, is detailed in Table 3. Among the microelements, the meal had the highest content of Mn at 8.77 mg/100 g, followed by Zn, Fe, and Cu, which ranged between 0.56 and 7.95 mg/100 g. Regarding macroelements, the meal was particularly rich in K at 945.68 mg/100 g and P at 636.01 mg/100 g. It also contained high levels of Ca at 410.09 mg/100 g and Mg at 205.58 mg/100 g. The high calcium levels in black soldier fly larvae result from a significant amount of calcium carbonate present in their exoskeletons. In comparison to the meal derived from dried yellow mealworms (*T. molitor*) [43], the meal of HI showed significantly higher levels of Mn, double the amount of Fe, and five times more Ca, while also having nearly half the P content. As for the black soldier fly larvae themselves, the content of individual elements varies widely. For example, the reported Ca content ranges from 120 mg to 3.57 g/100 g, while the P content ranges from 100 mg to 1.03 g/100 g, and the Fe from 10 mg to 19.10 g/100 g [51].

Mineral content variations in larvae can be linked to their growth stages. Liu et al. [52] found that early prepupae larvae contained significantly more Ca and nearly double the P content—620 mg per 100 g—compared to mature larvae, which had 350 mg per 100 g. This difference may result from cuticle formation during the prepupal stage. Meanwhile, mature larvae showed higher levels of Na, Fe, and Zn. The ash content of the nutrients consumed by larvae also affects the mineral composition of the meal, varying based on whether the larvae are fed plant waste, kitchen scraps, or animal feed [51]. For instance, larvae reared on cattle dung grow slower and are smaller than those fed poultry feed [53,54]. Moreover, the mineral content is influenced by processing methods, including blanching, freezing, drying, and grinding, and storage conditions. Environmental factors such as temperature, humidity, sunlight, and pH are also among the most important in the development and lifespan of larvae [51].

#### 3.2.2. Mineral Composition of Bread

The inclusion of black soldier fly larvae in bread has shown the potential of insects to enrich whole wheat bread with minerals (Table 3). We observed significant increases (*p* < 0.05) in all analyzed minerals, even at the lowest enrichment level of 10%. For microelements, the most notable increase was seen for Mn in the HI30 bread, which doubled compared to the HI0 bread. Similarly, the increases in Fe and Zn exceeded 50%. Among the macroelements, the most remarkable threefold increase was observed for Ca with the 30% addition of HI. The enrichment with insect meal at levels of 10% and 30% resulted in increases in Mg by 26% and 67%, respectively, while K and P increased by more than 10% and approximately 40%. The inclusion of larvae led to a significant increase in Na, although it did not exceed 10%. This is important, considering the widespread overconsumption of sodium among populations worldwide [55]. In contrast to a previous study on oatmeal cookies with dried yellow mealworm [43], black soldier fly proved more effective in enhancing bread, especially with calcium. For instance, mealworm additions resulted in Ca increases below 3%, and Fe of only 13%. These findings further highlight the potential of black soldier fly larvae to enhance the content of dietary micronutrients, offering a promising solution for developing nutrient-rich foods. However, it is essential to investigate the bioavailability of minerals in foods that include edible insects. This is important because certain antinutrients, such as oxalates, alkaloids, saponins, and chitin, can bind to minerals, hindering their absorption and reducing the nutritional value of insect-based products [56]. For instance, studies involving humans have shown that iron absorption from meals containing insects is low. One randomized trial indicated that the iron absorption rate from house crickets (*Acheta domesticus*) in iron-deficient women was only about 3.06–4.92%, compared to 14.2% from refined maize porridge [57]. This low bioavailability is attributed to inhibitors found in insect biomass, such as chitin and calcium, which negatively impact the absorption of both heme and non-heme iron.

### 3.3. Polyphenols and Antioxidant Properties

#### 3.3.1. TPC and Antioxidant Activity of Insect Meal

Insects can offer a novel and intriguing source of bioactive compounds. Among these, phenolic constituents, predominantly flavonoids, are a significant group that plays an important role in human nutrition. Insects acquire phenolic compounds by absorbing them from their diet and through a process called sclerotization, where they incorporate these compounds into the cuticular matrix with structural proteins and chitin [58]. The total phenolic content (TPC) in the larvae was calculated to be 81.79 mg GAE/100 g d. m. (Table 4), which is comparable to the TPC found in certain fruits, such as peach [59] or apricot [60]. This value falls within the range of 70–90 mg ChlA/100 g d.m. reported by Bogusz et al. [61]. However, this TPC is approximately ten times lower than that of the yellow mealworm [43].

Bioactive compounds, including polyphenols, significantly contribute to the antioxidant activity of food. Insects also serve as potential sources of bioactive peptides, proteins, vitamins, and free amino acids. Various amino acid sequences derived from insects have been recognized for their in vitro bioactive properties [62]. To assess antioxidant capacity, the photochemiluminescence (PCL) method was used to measure water-soluble (ACW) and lipid-soluble (ACL) components. The water-soluble fraction contained antioxidants, such as flavonoids, ascorbic acid, and amino acids. In contrast, the lipid-soluble fraction included tocopherols, tocotrienols, and other compounds. *H. illucens* exhibited an antioxidant value at 241.17 µmol TE/100 g d.m., with nearly 83% consisting of hydrophobic antioxidants. However, this antioxidant activity is significantly lower compared to *T. molitor* with 1190.05 µmol TE/100 g d.m. [43]. Other studies have confirmed this finding by comparing TPC and DPPH activity in various insect species. The highest DPPH scavenging activity was observed in *T. molitor* larvae (0.85 mg TEAC/g), while the lowest was found in *H. illucens* larvae (0.20 mg TEAC/g) [63].

#### 3.3.2. TPC and Antioxidant Activity of Bread

The TPC analysis revealed significantly higher levels of polyphenolic compounds in bread enriched with HI (Table 4). The TPC ranged from 88.61 to 97.16 mg GAE per 100 g d. m., with increases of 15.4% for HI10 and 26.5% for HI30. The observed increases are modest due to the low TPC in the HI meal used for fortification. Nonetheless, these findings support previous studies showing that insects enhance the phenolic and antioxidant content of bakery products. For example, muffins fortified with 2%, 6%, and 10% of *T. molitor* had TPC values up to six times higher than control muffins [21]. Additionally, gluten-free bread with 2%, 6%, and 10% of *A. domesticus* powder showed TPC values one to four times higher than control samples, emphasizing the positive impact of insect flour on cereal products [64].

Although *H. illucens* exhibits the lowest antioxidant activity among insects, the ACW method showed that bread with 10% HI increased antioxidant activity by 26% (*p* < 0.05), while further addition had no significant effect. In contrast, the ACL method indicated HI percentage-dependent increases in antioxidant activity, ranging from 5.7% to 13.7% (*p* < 0.05). Tocopherols are among the most important lipid-soluble antioxidants found in food and in human and animal tissues. Their potential health benefits include the prevention of certain cancers, heart disease, and other chronic conditions [65]. Black soldier fly larvae contain tocopherols, especially α-tocopherol, which can be bioaccumulated from their diet [66]. While tocopherol deficiencies are scarce, ongoing intake of tocopherols and tocotrienols from both common and novel dietary sources is beneficial. The overall antioxidant activity did not show a significant difference between the HI0 and HI10 samples, although there was a 10% increase in the HI10 sample. In contrast, bread containing 30% HI exhibited an antioxidant activity of 284.17 µmol TE/100 g, which represents an increase of 5% to 16% compared to the other two samples (*p* < 0.05). These are again relatively modest increases compared to other studies. For instance, Djouadi et al. [67] revealed that crackers enriched with 6% *T. molitor* flour displayed three times greater DPPH radical scavenging activity and four times higher iron-reducing capacity (FRAP) compared to control crackers. Additionally, Zielińska et al. [21] reported that flours derived from adult *Gryllodes sigillatus* and *T. molitor* larvae, when added at 10% increased the antioxidant capacity in muffins by eighteen times and five times, respectively. Another study shows that cricket powder improves the antioxidant activity of gluten-free protein bars [68]. The scavenging activity (%) for ABTS increased from 7.33 to 50.17, and for DPPH, it rose from 4.10 to 33.88 with 25% cricket powder. Therefore, the current results suggest that HI enhances the antioxidant activity of bread to a limited extent, primarily due to the lower activity of the larvae themselves, although it remains significant. Furthermore, Zielińska et al. [62] noted that the effectiveness of antioxidant-rich foods in vivo depends on their bioavailability. Thus, evaluating the in vitro antioxidant potential of insect-enriched products is crucial for future research on their in vivo activity.

### 3.4. Fatty Acid Composition

#### 3.4.1. Fatty Acid Profile of Insect Meal

The current study found that the fatty acid composition of the HI used for bread fortification primarily consisted of saturated fatty acids (SFAs), which made up 79.76% of the total fatty acids. Lauric acid (C12:0) was the most abundant, comprising 52.9%, followed by palmitic acid (C16:0) at 13.0% and myristic acid (C14:0) at 10.4% (Table 5). This suggests that *H. illucens* used in this study is a significant source of lauric acid, comparable to other major sources of C12:0, including coconut and palm oil, as well as breast milk [69]. Previous studies have shown that black soldier fly larvae have a higher level of saturated fatty acids than unsaturated, with the highest level of lauric acid, followed by palmitic acid, myristic acid, oleic acid (C18:1, cis9), and linoleic acid (C18:2(n-6)) [32,70,71,72]. Monounsaturated fatty acids (MUFAs) constituted 12.90%, with oleic acid being the most prevalent at 8.37%. Of note, this dietary fatty acid is the most important MUFA found in products like olive oil and meat, recognized for its beneficial health effects [73]. Polyunsaturated fatty acids (PUFAs) made up 7.08% of the total. The ratio of n-6 to n-3 fatty acids was found to be 12.28. The meal derived from *H. illucens* was characterized by a high level of hypercholesterolemic fatty acids (OFAs) at 78.0%, whereas hypocholesterolemic fatty acids (DFAs) were lower, at 21.7% of the total fatty acids.

#### 3.4.2. Fatty Acid Profile of Bread

In the control bread, MUFA were the most abundant, comprising 46.60% of the total fatty acid composition (Table 5). Oleic acid was the predominant MUFA, accounting for over 42%. PUFAs made up 30.93% of the fatty acid profile, with linoleic acid contributing more than 26% and linolenic acid (C18:3, n-3) constituting 4.8%. Meanwhile, this study showed that adding HI significantly increased the saturated fatty acid (SFA) content in bread. With 10% HI, the SFAs increased to 63.17% of total fatty acids, and with 30% HI, they further increased to 73.83% due to considerable amounts of lauric and myristic acids (*p* < 0.05). In contrast, the levels of palmitic and pentadecanoic (C15:0) acids significantly decreased (*p* < 0.05), as their amounts were higher in the original bread than in larvae. The addition of HI resulted in a significant reduction in both MUFAs and PUFAs (*p* < 0.05). In the HI10 bread, MUFAs comprised 22.1%, which is half the amount found in the original bread, while in the HI30 samples, it constituted 16.6% of the total fatty acid composition, primarily owing to the significant decrease in oleic acid. Interestingly, other MUFAs, such as myristoleic (C14:1) and palmitoleic (C16:1) acids, showed a significant increase (*p* < 0.05). Similarly, the HI0 bread had the highest level of PUFAs at 30.9%, whereas the HI10 and HI30 samples contained 14.7% and 9.5% PUFAs, respectively, mostly due to the decreasing levels of linoleic acid (*p* < 0.05). Notably, since bread was deficient in conjugated linoleic acid (CLA; C18:2 cis9 trans11), it increased in the formulated breads, despite initially being low in HI. While the overall benefits of CLA remain debatable, it is thought to contribute to reduced body fat, improved bone mass, and potential anti-cancer, anti-diabetic, and immune-modulating effects [77].

Nevertheless, the nutritional quality of lipids and their potential health effects can be assessed using various indicators, including the n-6/n-3 ratio, the Atherogenic Index (AI), the Thrombogenic Index (TI), and the Hypocholesterolemic/Hypercholesterolemic (H/H) ratios (Table 5). In the control bread (HI0), the n-6/n-3 ratio was 5.48. Adding HI significantly increased this ratio (*p* < 0.05) to 7.53 with 10% HI and 8.93 with 30% HI, due to a lower content of n-3 fatty acids. The human body can maintain optimal health with an n-6/n-3 intake ratio of 5:1, as excessive intake of n-6, especially without sufficient n-3s, can promote inflammation and metabolic issues [78]. Studies show that cookies with mealworms have a lower n-6/n-3 ratio [41], and Mihaly Cozmuta et al. [79] found similar results for sponge cakes. However, Kowalski et al. [22] reported an increased n-6/n-3 ratio for biscuits with mealworms. The AI, TI, and H/H ratios varied among breads. Lower AI and TI values are associated with reduced cardiovascular risk, while higher H/H levels are preferred [80]. Control bread (HI0) had the lowest AI and TI (0.32 and 0.39, respectively), while breads with insect meal showed significantly higher AI and TI (*p* < 0.05) and a lower H/H ratio (*p* < 0.05). Overall, adding insect meal to bread significantly decreased beneficial DFAs and increased harmful OFAs. This is because the nutritional value of insect fat is comparable to that of animal fats, which is characterized by a low ratio of PUFAs to SFAs and a high n-6 to n-3 fatty acid ratio [22]. When evaluating the nutritional value of insect fat, it is crucial to consider both the potential health benefits and the associated risks of saturated fatty acids. For example, lauric acid is essential for the integrity of cell membranes and plays a role in various biological processes, including signal transduction, growth, differentiation, and apoptosis, particularly within the central nervous and cardiovascular systems [81]. However, it is important to note that lauric acid has the most significant cholesterol-raising effect among all fatty acids, increasing levels of both low-density lipoprotein cholesterol (LDL-C) and high-density lipoprotein cholesterol (HDL-C). This is particularly relevant because cardiovascular diseases (CVD) are a leading cause of mortality worldwide, with dietary habits being a major risk factor for dyslipidemia. Saturated fatty acids can exacerbate CVD by worsening dyslipidemia, especially due to the cholesterol-raising effects of lauric acid. Research also suggests that lauric acid may trigger eryptosis, a novel mechanism related to CVD, which can guide dietary strategies for CVD prevention and management [82]. Meanwhile, recent studies have also provided valuable insights into the beneficial properties of C12:0. For instance, Srisuksai et al. [83] reveal that lauric acid from black soldier fly larvae oil has antioxidant properties. It also exhibits antibacterial, antiviral, antifungal, and anticancer effects [84,85,86]. Lauric acid is particularly effective against Gram-positive bacteria [32] and is widely used to produce surfactants in the food, cosmetic, shampoo, and pharmaceutical industries [86]. Palmitic and myristic acids are known to play important roles in signaling and immune responses. Palmitic acid, in particular, helps regulate hormone secretion [87]. Thus, SFAs may be beneficial or harmful depending on many factors, including the amount consumed and individual health factors. Instead of eliminating SFAs from the diet, individuals should focus on maintaining a diverse and balanced intake.

### 3.5. Texture Profile Analysis

The level of insect meal substitution in bakery products is crucial for achieving desirable textural qualities. Texture Profile Analysis (TPA), also referred to as the two-bite test or double compression test, simulates chewing and effectively assesses texture properties such as hardness, gumminess, springiness, chewiness, and adhesiveness [88,89].

The effect of black soldier fly larvae on bread was observed as an increase in hardness and a decrease in springiness and cohesiveness (*p* < 0.05); (Table 6). This may be attributed to the low water content relative to the rising levels of insect protein. In breads containing insect meal, the water content is likely insufficient to adequately hydrate the gluten proteins, which are crucial for forming a proper network, thus resulting in harder bread. Only free water acts as a plasticizer in dough, affecting the softening of the bread crumb [37]. Bawa et al. [90] and Bresciani et al. [91] enriched wheat bread with cricket flour at levels between 5% and 20%, reporting increased crumb hardness compared to their non-enriched counterparts. Mafu et al. [92] enriched whole wheat bread with cricket powder at levels of 10%, 15%, 20%, 25%, and 30%, also observing increased hardness compared to the non-enriched version. Others claim that this phenomenon may be due to the contribution of cricket flour to a higher fiber content (chitin) in the enriched bakery products [33].

Additionally, the inclusion of HI resulted in a reduction in bread crumb springiness, which decreased by 7% at a 10% inclusion level and by 47% at a 30% level. Springiness in bread crumbs refers to their ability to return to their original shape after being compressed, indicating a resilient and elastic texture [93]. The gluten network in bread dough plays a key role in maintaining this springiness. In turn, the cohesiveness of bread crumbs was reduced by 20% and 54% at 10% and 30% larva inclusion levels, respectively. This decreased cohesiveness reflects how well the crumb particles bond together, which in turn affects the bread’s dryness, brittleness, and tendency to fall apart [89]. Similarly, Siqueira Galvão Novo et al. [37] found that adding 15% black soldier fly larvae powder affected cohesiveness, as the bread was less cohesive compared to the control made from wheat flour. Likewise, Kowalski et al. [47] produced breads enriched with buffalo worm (*Alphitobius diaperinus*), cricket, and mealworm and observed a significant reduction in cohesiveness with a 20% replacement of wheat flour.

Notably, a 10% inclusion of insect meal resulted in only a slight and statistically insignificant increase (*p* > 0.05) in gumminess and chewiness. In contrast, a 30% inclusion led to significant increases in both parameters (*p* < 0.05). The observed decrease in gumminess (26%) and chewiness (39%) with the 30% HI could be attributed to factors such as insufficient fermentation, low hydration, and inadequate gluten development. These can prevent the dough from retaining gas produced during fermentation, resulting in bread that is under-risen and crumbly. On the other hand, the slight increase in gumminess and chewiness with the 10% HI may be linked to increased protein and fat content, as well as greater water absorption due to the higher protein levels in the dough. This higher hydration can contribute to a lighter and chewier crumb. Previous research supports these findings. For example, Bawa et al. [90] demonstrated that the chewiness of wheat bread increased when using cricket flour in amounts of 5%, 10%, and 15%. Similarly, Mafu et al. [92] reported a similar effect in whole wheat bread with cricket powder at levels of 10%, 15%, 20%, 25%, and 30%. However, García-Segovia et al. [94] found that the chewiness of breads enriched with 5% and 10% *T. molitor* powders decreased by 35.9% and 32.3%, respectively, compared to the control group. Ultimately, the significant results observed with a 30% inclusion of black soldier fly larvae are likely due to disruptions in the gluten network. Gluten-free, non-starch, and high-fiber ingredients can negatively impact bread-making by diluting the gluten and impeding the formation of the gluten network [95].

### 3.6. Color

Color is a key feature of bakery products and, alongside volume and texture, significantly influences consumer choice and acceptance [47]. The color of the loaf, particularly the crumb, impacts the acceptability of its organoleptic attributes. Various factors, including the chemical composition of ingredients, the color of raw materials, baking temperatures, and durations, affect the color changes in bakery products [33].

In this study, the color characteristics of both the crumb and crust of the breads were assessed, and the addition of HI had a significant effect on their color (*p* < 0.05); (Table 7). The lightness intensity (L*) of the bread’s crust ranged from 35.15 to 45.65, while the crumb ranged from 51.35 to 56.33. The red intensity (a*) was between 10.01 and 12.23 for the crust and 3.85 to 4.46 for the crumb. The yellow intensity (b*) ranged from 17.56 to 26.42 for the crust and 18.12 to 20.07 for the crumb. Chroma intensity (C*) varied from 20.24 to 28.90 for the crust and 18.52 to 20.57 for the crumb. Finally, hue intensity (h°) was between 60.21 and 66.13 for the crust and 77.46 to 78.42 for the crumb. As the proportion of HI increased, the L*, a*, and b* color parameters for both the crust and crumb decreased, indicating that the breads became darker, and the intensity of red and yellow colors diminished.

The decrease in the lightness of bakery products with increased insect powder substitution could be attributed to the enhanced Maillard reaction, which occurs due to the interaction between bioactive compounds and biomolecules—such as proteins and carbohydrates—from the insect powder that are released during baking [96]. In terms of the crust, the color change is highly dependent on the Maillard reaction, which occurs during baking between amino groups (e.g., amino acids and proteins) and carbonyl compounds (e.g., reducing sugars). This reaction contributes to the observed variations in the content of individual color components in the crust and crumb of the resulting breads. Increasing the proportion of raw materials with higher protein content can affect the intensity of the Maillard reaction [47].

Our findings are consistent with findings from other authors. For example, bread enriched with 5% HI powder showed a reduction in L* by 14% [49]. Bawa et al. [90] reported that enriching bread with cricket (*A. domesticus*) powder at 5%, 10%, and 15% resulted in decreases in L* values of 4.2%, 12.1%, and 14.1%, respectively. Similarly, using 10% and 20% cricket powder (*Gryllus assimilis*) in bread resulted in decreases in L* (7.5% and 14.6%) for the crust and crumb (25.8% and 35.4%, respectively); [97]. In research conducted by Siqueira Galvão Novo et al. [37], breads made with HI exhibited lower luminosity and higher a* values compared to wheat bread. These results partially correspond to Wrasiati et al. [38] and Draszanowska et al. [43], who showed that the brightness (L*) and yellowness (b*) of cookies decreased, while the redness (a*) increased with higher proportions of black soldier and mealworm flour. Conversely, Bottle et al. [98] observed that the addition of insect meals (*T. molitor* and *A. domesticus*) resulted in higher a* (redness) and b* (yellowness) values compared to the control. Additionally, research by Kowalski et al. [22,47] indicated that adding mealworm flour to sponge cake and bread formulations led to a decrease in lightness (L*) and an increase in redness (a*) and yellowness (b*).

In both the crust and crumb, increasing the content of HI led to a decrease in saturation (C*), resulting in a loss of intensity in the yellow color. The color hue (h°) of the bread crust decreased with increasing insect meal content, while the hue of the bread crumb increased, shifting towards a gray-yellow color. In addition, the bread crust exhibited a significantly lower brownness index (BI) in the HI30 sample (*p* < 0.05), while the bread crumb showed a slight, though not statistically significant, decrease (*p* > 0.05). In contrast, breads made with mealworm flours consistently displayed a higher BI value in the bread crumb, regardless of the quantity used [47].

### 3.7. Sensory Evaluation

Consumers are increasingly interested in nutrient-dense baked goods that look and taste appealing, as well as factors like food origin and environmental impact that can influence purchasing decisions [99]. Several studies [9,18,100] have explored consumer perceptions regarding the consumption of edible insects. These studies suggest that food neophobia associated with insects can be reduced if the insects are processed into powder and incorporated into bakery products. Therefore, food product developers should strive to find the right balance in using insect flour to maintain optimal organoleptic appeal [33].

In detail, bread containing 30% insect meal was rated less attractive overall, resulting in a crumb that scored below average in both porosity and elasticity (4.00 and 3.40 on a 10-point scale) (Table 8). Similarly, a study by Saraswati et al. [36] found that increasing the amount of larvae flour (5–20%) led to tortilla chips that were darker in color, which negatively affected their texture and crispness. In another study by Kowalski et al. [47], bread made with 30% cricket flour received significantly lower scores for appearance (4.19) compared to the control group, and a similar decrease in texture was noted. In the current study, bread containing 30% HI had significantly higher ratings for insect aroma (2.60) and insect taste (6.50). Interestingly, the ratings for foreign aroma and taste were low, at 0.70 and 1.80, respectively.

Although the aroma acceptability of bread containing the highest insect meal content was rated above average at 7.30, the taste acceptability was rated at 4.40. Similarly, Saraswati et al. [36] found that the addition of HI decreased the average panelists’ preference for the aroma and taste of the resulting larvae tortilla chips. However, when categorized by liking, the average hedonic test value for these tortilla chips was still rated as “like”. This indicates that the panelists generally appreciated the aroma and taste of black soldier fly larvae tortilla chips produced with 5–20% larvae. Kowalski et al. [47] observed significantly lower scores in the 5-point sensory evaluation for the aroma of breads containing 20% and 30% buffalo worm flour and 30% mealworm flour, scoring 3.65 and 3.71, respectively. The addition of insect flours (mealworm, buffalo worm, and cricket) at a 30% ratio significantly decreased the taste assessment scores of the bread (2.88, 2.32, and 3.81, respectively) compared to traditional wheat bread. Wrasiati et al. [38] reported that the formulation of HI (0–25%) did not significantly impact (*p* > 0.05) the aroma of the cookies. However, the highest average rating for aroma was observed in cookies without the additive, while cookies with 25% HI were rated the lowest. In addition, the highest average taste-liking scores were found in cookies without HI and those with 5% and 10% HI. Panelists noted that a flavor distinct from wheat flour began to emerge at the 15% addition, while cookies containing 25% HI received the worst ratings.

The overall acceptability of the bread with 30% HI was rated lower at 6.10 (*p* < 0.05) compared to 10% HI, which received a maximum rating of 8.90. However, this was not significantly different from HI0, which scored 8.80. These results largely align with Kowalski et al. [47], since the overall acceptability of bread with insect flours (mealworm, buffalo worm, cricket) was significantly lower for formulations containing 20% and 30%. Wrasiati et al. [38] also found that the highest average ratings for overall acceptance were for cookies made without the additive, as well as those with 5% and 10% larvae. Cookies with the highest formulation received the lowest average ratings. Saraswati et al. [36] recorded the overall acceptance of HI larvae tortilla chips, noting a significant decrease in acceptance as the additive increased from 5% to 20%.

Bread with a 10% addition of insect meal showed no significant difference (*p* > 0.05) in 90% of the tested sensory characteristics compared to those without the additive. These findings align with those of Kowalski et al. [47], which indicated that breads made with 10% insect flour (including mealworm, buffalo worm, and cricket) received favorable organoleptic acceptance from consumers based on appearance, smell, texture, taste, and overall acceptability. In a study by Wrasiati et al. [38], cookies made with 10% HI were found to have the best sensory properties. Both control bread and enriched bread with up to 10% cricket substitution showed no significant difference in overall acceptability, as reported by Bawa et al. [90]. Similarly, Mafu et al. [92] found no difference in overall liking between control bread and bread enriched with 10% cricket powder. Generally, bread with up to 10% insect meal and muffins, biscuits, crackers, or cookies with 5% insect powder were considered acceptable by consumers, according to Amoah et al. [33]. In conclusion, the sensory analysis reveals that bread with a 10% addition of insect meal was favorably received by the panelists, making this proportion recommended for the preparation of staple goods.

## 4. Conclusions

This study investigated the effects of substituting whole wheat flour with black soldier fly larvae on the quality of bread. The larvae meal was characterized by high protein and lipid content and demonstrated good microbiological safety. The enriched breads exhibited a higher protein content and an increase in lipid levels of up to six times, alongside significant increases in minerals, particularly calcium, of up to 3 times. The breads also showed a decreased ratio of polyunsaturated fatty acids (PUFAs) to saturated fatty acids (SFAs), with lauric acid being the most prevalent fatty acid. While there were increases in total phenolic content and antioxidant activity, these were relatively modest. A lower addition of larvae helped maintain the lightness, yellowness, chroma and chewiness of the crumb at levels comparable to the control bread. Sensory evaluations indicated no significant differences between the control bread and the bread containing 10% larvae. Therefore, this study recommends a supplementation level of 10%, which improves the overall nutritional profile while preserving desirable physical and sensory qualities. Further research is necessary to evaluate the potential allergenicity and toxicity of the product to ensure its safety. This research could also encourage further investigation into the in vivo activity of bakery products that incorporate insects. A sensory evaluation of the final product should also be conducted with a larger group of participants to assess consumer acceptability. This is crucial, especially due to the intensity of the taste from the insect flour. Ultimately, it could help increase exposure to and familiarity with insect-based products.

## Figures and Tables

**Table 1:** Formulation and coding of bread.

Ingredient (g)/1000 g	Formulation and Coding		
	(Ratio of HI *)		
	HI0 (0%)	HI10 (10%)	HI30 (30%)
Dried black soldier	0	100	300
Whole wheat flour	460	360	160
Wheat flour	240	240	240
Milk powder	8	8	8
Sugar	10	10	10
Salt	9	9	9
Baker’s yeast	4	4	4
Oil	10	10	10
Water	259	259	259

HI—*Hermetia illucens*. * The HI coefficient is calculated as the proportion of insect meal in the recipe, replacing whole wheat flour.

**Table 2:** Proximate composition of insect meal and bread formulated with dried black soldier fly larvae.

	Samples	% Water	% Crude Protein	% Crude Fat
Raw material	HI meal	1.98 ± 0.02	36.70 ± 1.84	47.70 ± 2.39
Bread	HI0	32.36 ± 0.20 ^a^	9.94 ± 0.06 ^a^	0.30 ± 0.06 ^a^
	HI10	32.61 ± 0.07 ^ab^	12.00 ± 0.07 ^b^	1.10 ± 0.01 ^b^
	HI30	33.03 ± 0.27 ^b^	15.57 ± 0.05 ^c^	1.81 ± 0.11 ^c^

HI—*Hermetia illucens*; values are expressed as means (*n* = 3) ± standard deviations. Mean values with different letters (a, b, c) in the column are statistically different (*p*-value < 0.05).

**Table 3:** Mineral composition of insect meal and bread formulated with dried black soldier fly larvae.

	Samples	Microelements (mg/100 g)				Macroelements (mg/100 g)				
		Cu	Mn	Fe	Zn	Mg	Ca	Na	K	P
Raw material	HI meal	0.56 ± 0.01	8.77 ± 0.30	7.72 ± 0.33	7.95 ± 0.79	205.58 ± 4.54	410.09 ± 10.39	74.48 ± 1.59	945.68 ± 24.81	636.01 ± 16.30
Bread	HI0	0.24 ± 0.00 ^a^	1.66 ± 0.00 ^a^	1.97 ± 0.02 ^a^	1.98 ± 0.02 ^a^	50.78 ± 0.52 ^a^	41.73 ± 1.31 ^a^	335.94 ± 4.51 ^a^	232.36 ± 0.57 ^a^	221.19 ± 0.50 ^a^
	HI10	0.25 ± 0.00 ^b^	2.22 ± 0.01 ^b^	2.46 ± 0.05 ^b^	2.38 ± 0.03 ^b^	64.37 ± 3.66 ^b^	71.16 ± 0.57 ^b^	365.99 ± 7.74 ^b^	263.87 ± 3.61 ^b^	247.02 ± 1.75 ^b^
	HI30	0.28 ± 0.00 ^c^	3.39 ± 0.03 ^c^	3.25 ± 0.02 ^c^	3.12 ± 0.02 ^c^	85.11 ± 3.87 ^c^	134.80 ± 1.93 ^c^	369.22 ± 4.46 ^b^	333.69 ± 2.73 ^c^	308.77 ± 3.38 ^c^

HI—*Hermetia illucens*; values are expressed as means (*n* = 3) ± standard deviation. Mean values with different lowercase letters (a, b, c) in the column are statistically different (*p*-value < 0.05).

**Table 4:** Total phenolic content (TPC) and antioxidant activity of the insect meal and bread formulated with dried black soldier fly larvae.

	Samples	TPC	ACW	ACL	PCL
		[mg GAE/ 100 g d. m.]	[µmol TE/100 g d. m.]		
Raw material	HI meal	81.79 ± 0.57	41.74 ± 0.91	199.43 ± 8.41	241.17 ± 7.58
Bread	HI0	76.78 ± 3.28 ^a^	53.95 ± 1.33 ^a^	190.95 ± 0.95 ^a^	244.90 ± 18.29 ^a^
	HI10	88.61 ± 1.07 ^b^	68.12 ± 3.06 ^b^	201.90 ± 6.05 ^b^	270.02 ± 8.18 ^a^
	HI30	97.16 ± 2.54 ^c^	67.03 ± 0.92 ^b^	217.14 ± 3.52 ^c^	284.17 ± 4.29 ^b^

HI—*Hermetia illucens*; GAE—gallic acid equivalent; d. m.—dry matter; TE- Trolox equivalent; PCL—sum of ACW and ACL. Values are expressed as means (*n* = 3) ± standard deviations. Mean values with different letters (a, b, c) in the column are statistically different (*p*-value < 0.05).

**Table 5:** Fatty acid profile of the insect meal and bread formulated with dried black soldier fly larvae.

	Raw Material		Bread	
Samples	HI Meal	HI0	HI10	HI30
Fatty Acids	% of the Total Detected Fatty Acids			
C8:0	n.d.	0.25 ± 0.02 ^c^	0.08 ± 0.00 ^b^	0.04 ± 0.00 ^a^
C10:0	1.32 ± 0.04	0.61 ± 0.02 ^a^	1.08 ± 0.01 ^b^	1.21 ± 0.02 ^c^
C12:0	52.90 ± 0.57	0.85 ± 0.01 ^a^	37.90 ± 0.31 ^b^	47.30 ± 0.34 ^c^
C14:0	10.42 ± 0.12	2.44 ± 0.02 ^a^	8.10 ± 0.00 ^b^	9.64 ± 0.03 ^c^
C14:1	0.37 ± 0.01	0.21 ± 0.01 ^a^	0.30 ± 0.02 ^b^	0.35 ± 0.00 ^c^
C15:0	0.06 ± 0.01	0.35 ± 0.00 ^c^	0.14 ± 0.02 ^b^	0.10 ± 0.00 ^a^
C16:0	13.04 ± 0.22	14.28 ± 0.10 ^b^	13.42 ± 0.06 ^a^	13.35 ± 0.13 ^a^
C16:1	3.89 ± 0.06	0.62 ± 0.04 ^a^	2.96 ± 0.01 ^b^	3.58 ± 0.03 ^c^
C17:0	0.08 ± 0.01	0.15 ± 0.01 ^c^	0.10 ± 0.00 ^b^	0.09 ± 0.00 ^a^
C17:1	0.05 ± 0.01	0.12 ± 0.02 ^b^	0.05 ± 0.01 ^a^	0.04 ± 0.00 ^a^
C18:0	1.73 ± 0.02	3.15 ± 0.01 ^c^	2.18 ± 0.02 ^b^	1.98 ± 0.01 ^a^
C18:1 cis9 (n-9)	8.37 ± 0.09	42.65 ± 0.12 ^c^	17.81 ± 0.11 ^b^	12.14 ± 0.09 ^a^
C18:1 cis11	0.17 ± 0.01	2.12 ± 0.05 ^c^	0.70 ± 0.00 ^b^	0.38 ± 0.00 ^a^
C18:2 (n-6)	6.04 ± 0.07	26.15 ± 0.06 ^c^	12.24 ± 0.08 ^b^	8.09 ± 0.06 ^a^
C18:3 (n-3)	0.51 ± 0.01	4.77 ± 0.01 ^c^	1.70 ± 0.01 ^b^	0.94 ± 0.01 ^a^
C18:2 cis9 trans11	0.26 ± 0.01	n.d.	0.19 ± 0.01 ^a^	0.21 ± 0.01 ^b^
C20:0	0.09 ± 0.01	0.39 ± 0.06 ^c^	0.17 ± 0.01 ^b^	0.13 ± 0.00 ^a^
C20:1	0.06 ± 0.01	0. 87± 0.02 ^c^	0.32 ± 0.01 ^b^	0.14 ± 0.03 ^a^
C22:0	0.12 ± 0.01	n.d.	n.d.	n.d.
C22:5 (n-6)	0.26 ± 0.01	n.d.	0.56 ± 0.02 ^b^	0.30 ± 0.01 ^a^
ΣSFAs	79.76 ± 0.25	22.48 ± 0.19 ^a^	63.17 ± 0.25 ^b^	73.83 ± 0.18 ^c^
ΣMUFAs	12.90 ± 0.16	46.60 ± 0.13 ^c^	22.14 ± 0.15 ^b^	16.64 ± 0.11 ^a^
ΣPUFAs	7.08 ± 0.09	30.93 ± 0.06 ^c^	14.69 ± 0.10 ^b^	9.54 ± 0.06 ^a^
n-6/n-3 ratio DFAs OFAs	12.28 ± 0.04 21.71 ± 0.26 78.03 ± 0.27	5.48 ± 0.01 ^a^ 80.68 ± 0.21 ^c^ 19.32 ± 0.21 ^a^	7.53 ± 0.04 ^b^ 39.01 ± 0.26 ^b^ 60.99 ± 0.27 ^b^	8.93 ± 0.08 ^c^ 28.15 ± 0.19 ^a^ 71.85 ± 0.19 ^c^
AI	5.46 ± 0.06	0.32 ± 0.00 ^a^	2.28 ± 0.02 ^b^	3.82 ± 0.03 ^c^
TI	2.24 ± 0.01	0.39 ± 0.00 ^a^	1.04 ± 0.00 ^b^	1.62 ± 0.00 ^c^
H/H ratio	0.20 ± 0.001	4.19 ± 0.04 ^c^	0.54 ± 0.01 ^b^	0.31 ± 0.00 ^a^

HI—*Hermetia illucens*; values are expressed as means (*n* = 3) ± standard deviation; n.d.—not detected. Mean values with different letters (a, b, c) in the row are statistically different (*p*-value < 0.05) according to the Duncan test. SFAs—saturated fatty acids; MUFAs—monounsaturated fatty acids; PUFAs—polyunsaturated fatty acids; DFAs—hypocholesterolemic fatty acids (ΣUFA + C18:0); OFAs—hypercholesterolemic fatty acids (ΣSFA-C18:0); AI (atherogenic index) = (C12:0 + 4 × C14:0 + C16:0)/(Σn-6 + Σn-3 + ΣMUFA) and TI (thrombogenic index) = (C14:0 + C16:0 + C18:0)/0.5 × ΣMUFA + 0.5 × Σn-6 + 3 × Σn-3 + (Σn-3/Σn-6); [74,75]; H/H (hypocholesterolemic/hypercholesterolemic ratio) = (C18:1 cis9 + C18:2 n-6 + C18:3 n-3)/(C12 + C14:0 + C16:0) [76].

**Table 6:** Texture characteristics of bread formulated with dried black soldier fly larvae.

Samples	Hardness [N]	Springiness [-]	Cohesiveness [-]	Gumminess [N]	Chewiness [J]
HI0	24.49 ± 1.65 ^a^	0.70 ± 0.01 ^c^	0.50 ± 0.02 ^c^	12.53 ± 1.35 ^b^	9.05 ± 1.19 ^b^
HI10	30.86 ± 3.64 ^b^	0.65 ± 0.02 ^b^	0.40 ± 0.02 ^b^	15.28 ± 3.66 ^b^	10.02 ± 3.33 ^b^
HI30	39.84 ± 3.93 ^c^	0.37 ± 0.08 ^a^	0.23 ± 0.03 ^a^	9.28 ± 2.18 ^a^	3.59 ± 1.53 ^a^

HI—*Hermetia illucens*; values are expressed as means (*n* = 10) ± standard deviation. Mean values with different letters (a, b, c) in the column are statistically different (*p*-value < 0.05).

**Table 7:** Color characteristics of bread formulated with dried black soldier fly larvae.

	Bread Crust					
Samples	L*	a*	b*	C*	h°	BI
HI0	45.65 ± 1.75 ^c^	11.70 ± 0.88 ^b^	26.42 ± 0.87 ^c^	28.90 ± 1.02 ^c^	66.13 ± 1.39 ^b^	101.71 ± 6.32 ^b^
HI10	41.19 ± 2.01 ^b^	12.23 ± 0.91 ^b^	21.76 ± 2.24 ^b^	24.97 ± 2.30 ^b^	60.59 ± 1.73 ^a^	94.51 ± 9.10 ^ab^
HI30	35.15 ± 1.79 ^a^	10.01 ± 0.96 ^a^	17.56 ± 1.60 ^a^	20.24 ± 1.45 ^a^	60.21 ± 3.34 ^a^	88.17 ± 4.95 ^a^
	**Bread Crumb**					
HI0	56.33 ± 1.50 ^b^	4.46 ± 0.29 ^b^	20.07 ± 0.39 ^b^	20.57 ± 0.40 ^b^	77.46 ± 0.76 ^a^	49.06 ± 2.06 ^a^
HI10	54.85 ± 2.09 ^b^	4.04 ± 0.34 ^a^	19.71 ± 0.50 ^b^	20.12 ± 0.52 ^b^	78.42 ± 0.87 ^b^	49.17 ± 3.43 ^a^
HI30	51.35 ± 1.41 ^a^	3.85 ± 0.24 ^a^	18.12 ± 0.23 ^a^	18.52 ± 0.23 ^a^	77.99 ± 0.74 ^ab^	48.21 ± 1.91 ^a^

HI—*Hermetia illucens*; values are expressed as means (*n* = 10) ± standard deviation. Mean values with different letters (a, b, c) in the column are statistically different (*p*-value < 0.05). Lightness (L*) and color (a*—redness, b*—yellowness). Chroma (C*) and hue (h°).

**Table 8:** The sensory quality of bread formulated with dried black soldier fly larvae.

Attributes		Samples		
		HI0	HI10	HI30
Overall appearance		9.20 ± 0.92 ^b^	8.80 ± 1.23 ^b^	6.00 ± 0.82 ^a^
Intensity of	insects aroma	0.20 ± 0.42 ^a^	1.10 ± 1.10 ^a^	2.60 ± 0.84 ^b^
	foreign aroma	0.10 ± 0.32 ^a^	0.20 ± 0.42 ^a^	0.70 ± 1.06 ^a^
Aroma acceptability		8.90 ± 1.29 ^b^	9.00 ± 0.94 ^b^	7.30 ± 0.95 ^a^
Crumb porosity		8.60 ± 0.84 ^b^	8.20 ± 0.79 ^b^	4.00 ± 0.82 ^a^
Crumb elasticity		8.80 ± 0.92 ^b^	8.50 ± 1.18 ^b^	3.40 ± 1.07 ^a^
Intensity of	insects taste	0.40 ± 0.52 ^a^	1.60 ± 1.17 ^b^	6.50 ± 1.27 ^c^
	foreign taste	0.20 ± 0.42 ^a^	0.70 ± 0.82 ^a^	1.80 ± 1.03 ^b^
Taste acceptability		9.00 ± 0.94 ^b^	8.90 ± 1.20 ^b^	4.40 ± 1.58 ^a^
Overall acceptability		8.80 ± 0.79 ^b^	8.90 ± 1.20 ^b^	6.10 ± 1.37 ^a^

HI*—Hermetia illucens*; values are expressed as means (*n* = 10) ± standard deviations. Mean values with different letters (a, b, c) in the row are statistically different (*p*-value < 0.05).

## Data Availability

The original contributions presented in the study are included in the article; further inquiries can be directed to the corresponding author.

## Supplementary Materials

The following supporting information can be downloaded at https://www.mdpi.com/article/10.3390/foods14213686/s1. Table S1. Nutritional value of insect meal (g/100 g). Table S2. Microbiological analysis of insect meal according to European regulations for insect species intended for human consumption. References [39,40,41,42,101,102,103,104,105,106,107,108] are cited in the Supplementary Materials.

## References

[1] Fraser E.D.G. (2020). The challenge of feeding a diverse and growing population. Physiol. Behav..

[2] Sadigov R. (2022). Rapid growth of the world population and its socioeconomic results. Sci. World J..

[3] Willett W., Rockström J., Loken B., Springmann M., Lang T., Vermeulen S., Garnett T., Tilman D., DeClerck F., Wood A. (2019). Food in the Anthropocene: The EAT-Lancet Commission on healthy diets from sustainable food systems. Lancet.

[4] Berggren A., Jansson A., Low M. (2018). Approaching ecological sustainability in the emerging insects as food industry. Trends. Ecol. Evol..

[5] Onwezen M.C., Bouwman E.P., Reinders M.J., Dagevos H. (2021). A systematic review on consumer acceptance of alternative proteins: Pulses, algae, insects, plant-based meat alternatives, and cultured meat. Appetite.

[6] Katayama N., Ishikawa Y., Takaoki M., Yamashita M., Nakayama S., Kiguchi K., Kok R., Wada H., Mitsuhashi J. (2007). Entomophagy: A key to space agriculture. Adv. Space Res..

[7] Premalatha M., Abbasi T., Abbasi T., Abbasi S.A. (2011). Energy-efficient food production to reduce global warming and ecodegradation: The use of edible insects. Renew. Sustain. Energy Rev..

[8] Nijdam D., Rood T., Westhoek H. (2012). The price of protein: Review of land use and carbon footprints from life cycle assessments of animal food products and their substitutes. Food Policy.

[9] Herbert M., Beacom E. (2021). Exploring consumer acceptance of insect-based snack products in Ireland. J. Food Prod. Mark..

[10] Siddiqui S.A., Bahmid N.A., Mahmud C.M.M., Boukid F., Lamri M., Gagaoua M. (2022). Consumer acceptability of plant-, seaweed-, and insect-based foods as alternatives to meat: A critical compilation of a decade of research. Crit. Rev. Food Sci. Nutr..

[11] Tian H., Chen J. (2025). Association of food neophobia and food disgust with the willingness, benefits, and risks of insect food consumption among Chinese university students. Front. Nutr..

[12] Hopkins I., Farahnaky A., Gill H., Danaher J., Newman L.P. (2023). Food neophobia and its association with dietary choices and willingness to eat insects. Front. Nutr..

[13] Woolfa E., Zhua Y., Emoryb K., Zhaoc J., Liu C. (2019). Willingness to consume insect-containing foods: A survey in the United States. LWT-Food Sci. Technol..

[14] Sogari G., Riccioli F., Moruzzo R., Menozzi D., Sosa D.A.T., Li J., Liu A., Mancini S. (2023). Engaging in entomophagy: The role of food neophobia and disgust between insect and non-insect eaters. Food Qual. Prefer..

[15] Poortvliet P.M., Van Der Pas L., Mulder B.C., Fogliano V. (2019). Healthy, but disgusting: An investigation into consumers’ willingness to try insect meat. J. Econ. Entomol..

[16] Eckl M.R., Biesbroek S., Van’t Veer P., Geleijnse J.M. (2021). Replacement of meat with non-meat protein sources: A review of the drivers and inhibitors in developed countries. Nutrients.

[17] Sun-Waterhouse D., Waterhouse G.I.N., You L., Zhang J., Liu Y., Ma L., Gao J., Dong Y. (2016). Transforming insect biomass into consumer wellness foods: A review. Food Res. Int..

[18] Orkusz A., Wolanska W., Harasym J., Piwowar A., Kapelko M. (2020). Consumers’ attitudes facing entomophagy: Polish case perspectives. Int. J. Environ. Res. Public Health.

[19] Kourkouta L., Koukourikos K., Iliadis C., Ouzounakis P., Monios A., Tsaloglidou A. (2017). Bread and health. J. Pharm. Pharmacol..

[20] Roncolini A., Milanović V., Cardinali F., Osimani A., Garofalo C., Sabbatini R., Clementi F., Pasquini M., Mozzon M., Foligni R. (2019). Protein fortification with mealworm (*Tenebrio molitor* L.) powder: Effect on textural, microbiological, nutritional and sensory features of bread. PLoS ONE.

[21] Zielińska E., Pankiewicz U., Sujka M. (2021). Nutritional, physiochemical, and biological value of muffins enriched with edible insects flour. Antioxidants.

[22] Kowalski S., Mikulec A., Skotnicka M., Mickowska B., Makarewicz M., Sabat R., Gurgul A.W., Mazurek A. (2022). Effect of the addition of edible insect flour from yellow mealworm (*Tenebrio molitor*) on the sensory acceptance, and the physicochemical and textural properties of sponge cake. Pol. J. food Nutr. Sci..

[23] Duda A., Adamczak J., Chelminska P., Juszkiewicz J., Kowalczewski P. (2019). Quality and nutritional/textural properties of durum wheat pasta enriched with cricket powder. Foods.

[24] Petrescu-Mag R.M., Kopaei H.R., Petrescu D.C. (2022). Consumers’ acceptance of the first novel insect food approved in the European Union: Predictors of yellow mealworm chips consumption. Food Sci. Nutr..

[25] Bessa L.W., Pieterse E., Marais J., Hoffman L.C. (2020). Why for feed and not for human consumption? The black soldier fly larvae. Compr. Rev. Food. Sci. Food. Saf..

[26] Kaczor M., Bulak P., Proc-Pietrycha K., Kirichenko-Babko M., Bieganowski A. (2023). The Variety of Applications of *Hermetia illucens* in Industrial and Agricultural Areas—Review. Biology.

[27] Wang Y.S., Shelomi M. (2017). Review of black soldier fly (*Hermetia illucens*) as animal feed and human food. Foods.

[28] Lestari A., Wahyuni T.H., Mirwandhono E., Ginting N. (2020). Maggot black soldier fly (*Hermetia illucens*) nutritional content using various culture media. J. Integr. Anim. Sci..

[29] Gao Z., Wang W., Lu X., Zhu F., Liu W., Wang X., Lei C. (2019). Bioconversion performance and life table of black soldier fly (*Hermetia illucens*) on fermented maize straw. J. Clean. Prod..

[30] Chia S.Y., Tanga C.M., Osuga I.M., Cheseto X., Ekesi S. (2020). Nutritional composition of black soldier fly larvae feeding on agro-industrial by-products. Entomol. Exp. Appl..

[31] Ferdousi L., Sultana N., Bithi U.H., Lisa S.A., Hasan R., Siddique A.B. (2022). Nutrient profile of wild black soldier fly (*Hermetia illucens*) prepupae reared on municipal dustbin’s organic waste substrate. Proc. Natl. Acad. Sci. India Sect. B Biol. Sci..

[32] Rabani V., Cheatsazan H., Davani S. (2019). Proteomics and lipidomics of black soldier fly (Diptera: Stratiomyidae) and blow fly (Diptera: Calliphoridae) larvae. J. Insect Sci..

[33] Amoah I., Cobbinah J.C., Yeboah J.A., Essiam F.A., Lim J.J., Tandoh M.A., Rush E. (2023). Edible insect powder for enrichment of bakery products—A review of nutritional, physical characteristics and acceptability of bakery products to consumers. Future Foods.

[34] Ardoin R., Marx B.D., Boeneke C., Prinyawiwatkul W. (2021). Effects of cricket powder on selected physical properties and US consumer perceptions of whole-wheat snack crackers. Int. J. Food Sci. Technol..

[35] Defloor I., Nys M., Delcour J.A. (1993). Wheat-starch, cassava starch, and cassava flour impairment of the breadmaking potential of wheat-flour. Cereal Chem..

[36] Saraswati I.G.A.K.W., Putra I.G.A.M., Wrasiati L.P. (2021). The effect of adding black soldier fly (BSF) larvae (*Hermetia illucens* L.) flour on the characteristics of tortilla chips. Int. J. Curr. Microbiol. Appl. Sci..

[37] Siqueira Galvão Novo G., César Tondo E., Cruz Silveira Thys R., Cladera F. (2024). Production of protein-enriched bread through the incorporation of the black soldier fly (*Hermetia illucens*) larvae. Food Sci. Technol..

[38] Wrasiati L.P., Putra I.G.A.M., Yuarini D.A.A., Saraswati I.G.A.K.W. (2025). Development of nutrient-rich cookies using black soldier fly (BSF) flour. J. Insects Food Feed..

[39] AOAC (2005). Official Methods of Analysis Method (2005a) 934.01.

[40] AOAC (2005). Official Methods of Analysis Method (2005b) 2001.11.

[41] AOAC (2006). Official Methods of Analysis Method (2006) 991.36.

[42] AOAC (2005). Official Methods of Analysis Method (2005c) 945.16.

[43] Draszanowska A., Kurp L., Starowicz M., Paszczyk B., Czarnowska-Kujawska M., Olszewska M.A. (2024). Effect of the Addition of Yellow Mealworm (*Tenebrio molitor*) on the Physicochemical, Antioxidative, and Sensory Properties of Oatmeal Cookies. Foods.

[44] Starowicz M., Arpaci S., Topolska J., Wronkowska M. (2021). Phytochemicals and antioxidant activity in oat-buckwheat. Molecules.

[45] Christie W.W. (1973). Lipid Analysis; Isolation, Separation, Identification, and Structural Analysis of Lipids.

[46] Żegarska Z., Jaworski J., Borejszo Z. (1991). Evaluation of the Peisker modified method for extracting methyl esters from fatty acids. Acta Acad. Agri. Techn. Olst..

[47] Kowalski S., Mikulec A., Mickowska B., Skotnicka M., Mazurek A. (2022). Wheat bread supplementation with various edible insect flours. Influence of chemical composition on nutritional and technological aspects. LWT.

[48] (2012). Sensory Analysis—General Guidelines for the Selection, Training and Monitoring of Selected Assessors and Expert Sensory Assessors.

[49] Gonzalez C.M., Garzon R., Rosell C.M. (2019). Insects as ingredients for bakery goods. A comparison study of *H. illucens*, *A. domestica* and *T. molitor* flours. Innov. Food. Sci. Emerg. Technol..

[50] Nandiyanto A.B.D., Ragadhita R., Ana A., Hammouti B. (2022). Effect of starch, lipid, and protein components in flour on the physical and mechanical properties of Indonesian biji ketapang cookies. Int. J. Technol..

[51] Lu S., Taethaisong N., Meethip W., Surakhunthod J., Sinpru B., Sroichak T., Archa P., Thongpea S., Paengkoum S., Purba R.A.P. (2022). Nutritional Composition of Black Soldier Fly Larvae (*Hermetia illucens* L.) and Its Potential Uses as Alternative Protein Sources in Animal Diets: A Review. Insects.

[52] Liu X., Chen X., Wang H., Yang Q., ur Rehman K., Li W., Cai M., Li Q., Mazza L., Zhang J. (2017). Dynamic changes of nutrient composition throughout the entire life cycle of black soldier fly. PLoS ONE.

[53] Diener S., Zurbrügg C., Tockner K. (2009). Conversion of organic material by black soldier fly larvae: Establishing optimal feeding rates. Waste Manag. Res..

[54] Myers H.M., Tomberlin J.K., Lambert B.D., Kattes D. (2014). Development of black soldier fly (Diptera: Stratiomyidae) larvae fed dairy manure. Environ. Entomol..

[55] World Health Organization (2015). Global Status Report on Noncommunicable Diseases 2014.

[56] Li M., Mao C., Li X., Jiang L., Zhang W., Li M., Liu H., Fang Y., Liu S., Yang G. (2023). Edible Insects: A New Sustainable Nu-tritional Resource Worth Promoting. Foods.

[57] Mwangi M.N., Oonincx D.G.A.B., Hummel M., Utami D.A., Gunawan L., Veenenbos M., Zeder C., Cercamondi C.I., Zim-mermann M.B., van Loon J.J.A. (2022). Absorption of iron from edible house crickets: A randomized crossover stable-isotope study in humans. Am. J. Clin. Nutr..

[58] Herdeiro F.M., Carvalho M.O., Nunes M.C., Raymundo A. (2024). Development of healthy snacks incorporating meal from *Tenebrio molitor* and *Alphitobius diaperinus* using 3D printing technology. Foods.

[59] Li Y., Li L., Zhang X., Mu Q., Tian J., Yan J., Guo L., Wang Y., Song L., Yu X. (2023). Differences in Total Phenolics, Antioxidant Activity and Metabolic Characteristics in Peach Fruits at Different Stages of Ripening. LWT.

[60] Fan X., Jiao W., Wang X., Cao J., Jiang W. (2018). Polyphenol Composition and Antioxidant Capacity in Pulp and Peel of Apricot Fruits of Various Varieties and Maturity Stages at Harvest. Int. J. Food Sci. Technol..

[61] Bogusz R., Bryś J., Onopiuk A., Rybak K., Witrowa-Rajchert D., Nowacka M. (2023). Effect of Pulsed Electric Field Technology on the Composition and Bioactive Compounds of Black Soldier Fly Larvae Dried with Convective and Infrared–Convective Methods. Molecules.

[62] Zielińska E., Baraniak B., Karaś M. (2018). Identification of antioxidant and anti-inflammatory peptides obtained by simulated gastrointestinal digestion of three edible insects species (*Gryllodes sigillatus*, *Tenebrio molitor*, *Schistocerca gragaria*). Int. J. Food Sci. Technol..

[63] Džima M., Ivanišová E., Gálik B., Šimko M., Rolinec M., Hanušovský O., Kapusniaková M., Madajová V., Bíro D., Juráček M. (2025). Antioxidant activity and total polyphenol content of insects used as feed and food. J. Microbiol. Biotechnol. Food Sci..

[64] Kowalczewski P.Ł., Gumienna M., Rybicka I., Górna B., Sarbak P., Dziedzic K., Kmiecik D. (2021). Nutritional Value and Biological Activity of Gluten-Free Bread Enriched with Cricket Powder. Molecules.

[65] Shahidi F., de Camargo A.C. (2016). Tocopherols and Tocotrienols in Common and Emerging Dietary Sources: Occurrence, Applications, and Health Benefits. Int. J. Mol. Sci..

[66] Morand-Laffargue L., Vairo D., Halimi C., Chiarello E., Creton B., Sabatier D., Borel P. (2023). Ability of black soldier fly larvae to bioaccumulate tocopherols from different substrates and measurement of larval tocopherol bioavailability in vitro. J. Insects Food Feed.

[67] Djouadi A., Sales J.R., Carvalho M.O., Raymundo A. (2022). Development of Healthy Protein-Rich Crackers Using *Tenebrio molitor* Flour. Foods.

[68] Pecova M., Tauferova A., Pospiech M., Bartlova M., Tremlova B. (2023). Evaluation of gluten-free bars made with house cricket (*Acheta domesticus*) powder. J. Microbiol. Biotechnol. Food Sci..

[69] Dayrit F.M. (2015). The properties of lauric acid and their significance in coconut oil. J. Am. Oil Chem. Soc..

[70] Ebeneezar S., Tejpal C.S., Jeena N.S., Summaya R., Chandrasekar S., Sayooj P., Vijayagopal P. (2021). Nutritional evaluation, bioconversion performance and phylogenetic assessment of black soldier fly (*Hermetia illucens*, Linn. 1758) larvae valorized from food waste. Environ. Technol. Innov..

[71] Franco A., Scieuzo C., Salvia R., Petrone A.M., Tafi E., Moretta A., Schmitt E., Falabella P. (2021). Lipids from *Hermetia illecens*, an innovative and sustainable source. Sustainability.

[72] Almeida C., Murta D., Nunes R., Baby A.R., Fernandes A., Barros L., Rijo P., Rosado C. (2022). Characterization of lipid extracts from the *Hermetia illucans* larvae and their bioactivities for potential use as pharmaceutical and cosmetic ingredients. Heliyon.

[73] Flori L., Donnini S., Calderone V., Zinnai A., Taglieri I., Venturi F., Testai L. (2019). The nutraceutical value of olive oil and its bioactive constituents on the cardiovascular system. Focusing on main strategies to slow down its quality decay during production and storage. Nutrients.

[74] Ulbricht T.L.V., Southgate D.A.T. (1991). Coronary heart disease: Seven dietary factors. Lancet.

[75] Osmari E.K., Cecato U., Macedo F.A.F., Souza N.E. (2011). Nutritional quality indices of milk fat from goats on diets supplemented with different roughages. Small Rumin. Res..

[76] Ivanova A., Hadzhinikolova L. (2015). Evaluation of nutritional quality of common carp (*Cyprinus carpio* L.) lipidsthrough fatty acid ratios and lipid indices. Bulg. J. Agric. Sci..

[77] Badawy S., Liu Y., Guo M., Liu Z., Xie C., Marawan M.A., Ares I., Lopez-Torres B., Martínez M., Maximiliano J.-E. (2023). Conjugated linoleic acid (CLA) as a functional food: Is it beneficial or not?. Food Res. Int..

[78] Bishehkolaei M., Pathak Y. (2024). Influence of omega n-6/n-3 ratio on cardiovascular disease and nutritional interventions. Hum. Nutr. Metab..

[79] Mihaly Cozmuta A., Uivarasan A., Peter A., Nicula C., Kovacs D.E., Mihaly Cozmuta L. (2023). Yellow Mealworm (*Tenebrio molitor*) Powder Promotes a High Bioaccessible Protein Fraction and Low Glycaemic Index in Biscuits. Nutrients.

[80] Chen J., Liu H. (2020). Nutritional indices for assessing fatty acids: A mini-review. Int. J. Mol. Sci..

[81] Verma P., Ghosh A., Ray M., Sarkar S. (2020). Lauric acid modulates cancer-associated microRNA expression and inhibits the growth of the cancer cell. Anticancer Agents Med. Chem..

[82] Alfhili M.A., Aljuraiban G.S. (2021). Lauric Acid, a Dietary Saturated Medium-Chain Fatty Acid, Elicits Calcium-Dependent Eryptosis. Cells.

[83] Srisuksai K., Limudomporn P., Kovitvadhi U., Thongsuwan K., Imaram W., Lertchaiyongphanit R., Sareepoch T., Kovitvadhi A., Fungfuang W. (2024). Physicochemical properties and fatty acid profile of oil extracted from black soldier fly larvae (*Hermetia illucens*). Vet. World.

[84] Nakatsuji T., Kao M.C., Fang J., Zouboulis C.C., Zhang L., Gallo R.L., Huang C. (2009). Antimicrobial property of lauric acid against Propionibacterium acnes: Its therapeutic potential for inflammatory acne vulgaris. J. Investig. Dermatol..

[85] Anzaku A.A., Akyala J.I., Juliet A., Obianuju C. (2017). Antibacterial activity of lauric acid on some selected clinical isolates. Ann. Clin. Lab. Res..

[86] Suryati T., Julaeha E., Farabi K., Ambarsari H., Hidayat A.T. (2023). Lauric acid from the black soldier fly (*Hermetia illucens*) and its potential applications. Sustainability.

[87] Radzikowska U., Rinaldi A.O., Çelebi Z.C., Karaguzel D., Wojcik M., Cypryk K., Akdis M., Akdis C.A., Sokolowska M. (2019). The influence of dietary fatty acids on immune responses. Nutrients.

[88] Nishinari K., Kohyama K., Kumagai H., Funami T., Bourne M.C. (2013). Parameters of texture profile analysis. Food Sci. Technol. Res..

[89] Srilakshmi A. (2020). Texture Profile Analysis of Food and TPA Measurements: A Review Article. Int. J. Eng. Res. Technol..

[90] Bawa M., Songsermpong S., Kaewtapee C., Chanput W. (2020). Nutritional, sensory, and texture quality of bread and cookie enriched with house cricket (*Acheta domesticus*) powder. J. Food Process. Preserv..

[91] Bresciani A., Cardone G., Jucker C., Savoldelli S., Marti A. (2022). Technological performance of cricket powder (*Acheta domesticus* L.) in wheat-based formulations. Insects.

[92] Mafu A., Ketnawa S., Phongthai S., Schonlechner R., Rawdkuen S. (2022). Whole wheat bread enriched with cricket powder as an alternative protein. Foods.

[93] Trinh K.T., Glasgow S. On the texture profile analysis test. Proceedings of the Chemeca.

[94] García-Segovia P., Igual M., Martinez-Monzo J. (2020). Physicochemical properties and consumer acceptance of bread enriched with alternative proteins. Foods.

[95] Villarino C.B.J., Jayasena V., Coorey R., Chakrabarti-Bell S., Johnson S.K. (2016). Nutritional, health, and technological functionality of lupin flour addition to bread and other baked products: Benefits and challenges. Crit. Rev. Food Sci. Nutr..

[96] Ribas-Agusti A., Martin-Belloso O., Soliva-Fortuny R., Elez-Martinez P. (2017). Food processing strategies to enhance phenolic compounds bioaccessibility and bioavailability in plant-based foods. Crit. Rev. Food Sci. Nutr..

[97] Da Rosa Machado C., Thys R.C.S. (2019). Cricket powder (*Gryllus assimilis*) as a new alternative protein source for gluten-free breads. Innov. Food Sci. Emerg. Technol..

[98] Bottle E., Espinosa-Ramírez J., Serna-Saldívar S.O., Tejada-Ortigoza V. (2024). Effect of full fat and defatted insect meals in breadmaking quality. LWT.

[99] Alemu M.H., Olsen S.B., Vedel S.E., Kinyuru J.N., Pambo K.O. (2017). Can insects increase food security in developing countries? An analysis of Kenyan consumer preferences and demand for cricket flour buns. Food Secur..

[100] Wilkinson K., Muhlhausler B., Motley C., Crump A., Bray H., Ankeny R. (2018). Australian consumers’ awareness and acceptance of insects as food. Insects.

[101] AOAC (1985). Official Methods of Analysis Method (1985) 985.29.

[102] AOAC (2022). Official Methods of Analysis Method (2022) 940.26.

[103] European Commission (2022). Commission Implementing Regulation (EU) 2022/169 of 8 February 2022 Authorising the Placing on the Market of Frozen, Dried and Powder Forms of Yellow Mealworm (*Tenebrio molitor* Larva) as a Novel Food Under Regulation (EU) 2015/2283 of the European Parliament and of the Council, and amending Commission Implementing Regulation (EU) 2017/2470. European Commission. http://data.europa.eu/eli/reg_impl/2022/169/oj.

[104] (2013). Microbiology of the Food Chain—Horizontal Method for the Enumeration of Microorganisms.

[105] (2008). Microbiology of Food and Animal Feeding Stuffs—Horizontal Method for the Enumeration of Yeasts and Moulds.

[106] (2017). Microbiology of the Food Chain—Horizontal Method for the Detection and Enumeration of Enterobacteriaceae.

[107] (2004). Plate Method (Depth Plating).

[108] (2017). Microbiology of the Food Chain—Horizontal Method for the Detection, Enumeration and Serotyping of Salmonella.

