# Supplementary material for: The Potential of Black Soldier Fly (Hermetia illucens L.) Larvae in Whole Wheat Bread Production: Effects on Physicochemical, Antioxidative, and Sensory Properties
Source: Foods. 2025 Oct 29;14(21):3686. doi: 10.3390/foods14213686 (PMC12607490; doi:10.3390/foods14213686)
Supplement: Supplementary file 1 [file foods-14-03686-s001.zip › foods-3921148-supplementary.pdf]

Supplementary Materials

Table S1. Nutritional value of insect meal (g/100g).

Parameter	Result
moisture	1.98±0.02
protein	36.70±1.84
fat	47.70±2.39
dietary fiber	5.71±1.14
ash	3.75±0.11

Chemical Composition of Insect Meal

The insect meal was analyzed following the procedures established by the Association of Official Analytical Chemists. The moisture content was as outlined in AOAC procedure No. 934.01 [39]. Crude protein content was assessed using the Kjeldahl method (AOAC No. 2001.11); [40]. Crude fat content was measured using the Soxhlet method (AOAC No. 991.36); [41] with pure petroleum ether (No. 945.16); [42]. Dietary fiber was measured using the AOAC method No. 985.29 [101], and ash content was assessed using a separate standardized method (AOAC No. 940.26)[102].

Table S2. Microbiological analysis of insect meal according to European regulations for insect species intended for human consumption.

Microbiological criteria	Black soldier fly larvae	EU Regulation [103]
Total aerobic colony count	3.8×10^2 CFU/g	$\leq 10^5$
Yeasts and molds	$<1.0 \times 10^1$ CFU/g	$\leq 10^2$
Enterobacteriaceae	$<1.0 \times 10^1$ CFU/g	$\leq 10^2$
Escherichia coli	$<1.0 \times 10^1$ CFU/g	≤50
Salmonella spp.	Not detected in 25 g	Not detected in 25 g

Microbiological Analysis of Insect Mean

The following microbiological analyses were conducted in black soldier fly larvae: total aerobic colony count [104], yeasts and molds [105], *Enterobacteriaceae* [106], *Escherichia coli* [107], and *Salmonella* spp. [108].

References

39. AOAC. *Official methods of analysis method (2005a) 934.01,* 18th Ed., AOAC International, Gaithersburg, MD, USA, 2005.

40. AOAC. *Official methods of analysis method (2005b) 2001.11,* 18th Ed., AOAC International, Gaithersburg, MD, USA, 2005.

41. AOAC. *Official methods of analysis method (2006) 991.36,* 18th Ed., AOAC International, Gaithersburg, MD, USA, 2006.

42. AOAC. *Official methods of analysis method (2005c) 945.16,* 18th Ed., AOAC International, Gaithersburg, MD, USA, 2005.

101. AOAC. *Official methods of analysis method (1985) 985.29,* 18th Ed., AOAC International, Gaithersburg, MD, USA, 1985.

102. AOAC. *Official methods of analysis method (2022) 940.26,* 21st Ed., AOAC International, Gaithersburg, MD, USA, 2022.

103. European Commission (2022). Commission Implementing Regulation (EU) 2022/169 of 8 February 2022 authorising the placing on the market of frozen, dried and powder forms of yellow mealworm (Tenebrio molitor larva) as a novel food under Regulation (EU) 2015/2283 of the European Parliament and of the Council, and amending Commission Implementing Regulation (EU) 2017/2470. European Commission. Retrieved from http://data.europa.eu/eli/reg_impl/2022/169/oj (accessed on 18 September 2025).
104. ISO 4833-1:2013; Microbiology of the food chain — Horizontal method for the enumeration of microorganisms. ISO: Geneva, Switzerland, 2013.
105. ISO 21527-2:2008; Microbiology of food and animal feeding stuffs — Horizontal method for the enumeration of yeasts and moulds. ISO: Geneva, Switzerland, 2008.
106. ISO 21528-2:2017; Microbiology of the food chain — Horizontal method for the detection and enumeration of *Enterobacteriaceae*. ISO: Geneva, Switzerland, 2017.
107. PN-ISO 16649-2:2004; Plate method (depth plating). Polish Committee for Standardization: Warsaw, Poland, 2004.
108. ISO 6579-1:2017; Microbiology of the food chain — Horizontal method for the detection, enumeration and serotyping of *Salmonella*. ISO: Geneva, Switzerland, 2017.
